# Volumetric modulated arc therapy versus intensity-modulated proton therapy in neoadjuvant irradiation of locally advanced oesophageal cancer

**DOI:** 10.1186/s13014-020-01570-y

**Published:** 2020-05-24

**Authors:** Eren Celik, Wolfgang Baus, Christian Baues, Wolfgang Schröder, Alessandro Clivio, Antonella Fogliata, Marta Scorsetti, Simone Marnitz, Luca Cozzi

**Affiliations:** 1 Department of Radiation Oncology and Cyberknife Center, Faculty of Medicine and University Hospital Cologne, University of Cologne, Cologne, Germany; 2 Department of General, Visceral, Cancer and Transplantation Surgery, Faculty of Medicine and University Hospital Cologne, University of Cologne, Cologne, Germany; 3 Zentrum für Radiotherapie, Rüti, Switzerland; 4 Radiotherapy and Radiosurgery Department, Humanitas Clinical and Research Center, IRCSS, Via Manzoni 56, 20089 Milan-Rozzano, Italy; 5 Department of Biomedical Sciences, Humanitas University, Milan-Rozzano, Italy

**Keywords:** Intensity-modulated proton therapy, VMAT, RapidArc, Oesophagal cancer, Secondary cancer risk estimate

## Abstract

**Background:**

To investigate the role of intensity-modulated proton therapy (IMPT) compared to volumetric modulated arc therapy (VMAT), realised with RapidArc and RapidPlan methods (RA_RP) for neoadjuvant radiotherapy in locally advanced oesophagal cancer.

**Methods:**

Twenty patients were retrospectively planned for IMPT (with two fields, (IMPT_2F) or with three fields (IMPT_3F)) and RA_RP and the results were compared according to dose-volume metrics. Estimates of the excess absolute risk (EAR) of secondary cancer induction were determined for the lungs. For the cardiac structures, the relative risk (RR) of coronary artery disease (CAD) and chronic heart failure (CHF) were estimated.

**Results:**

Both the RA_RP and IMPT approached allowed to achieve the required coverage for the gross tumour volume, (GTV) and the clinical and the planning target volumes, CTV and PTV (V_98%_ > 98 for CTV and GTV and V_95%_ > 95 for the PTV)). The conformity index resulted in 0.88 ± 0.01, 0.89 ± 0.02 and 0.89 ± 0.02 for RA_RP, IMPT_2F and IMPT_3F respectively. With the same order, the homogeneity index for the PTV resulted in 5.6 ± 0.6%, 4.4 ± 0.9% and 4.5 ± 0.8%. Concerning the organs at risk, the IMPT plans showed a systematic and statistically significant incremental sparing when compared to RA_RP, especially for the heart. The mean dose to the combined lungs was 8.6 ± 2.9 Gy for RA_RP, 3.2 ± 1.5 Gy and 2.9 ± 1.2 Gy for IMPT_2F and IMPT_3F. The mean dose to the whole heart resulted to 9.9 ± 1.9 Gy for RA_RP compared to 3.7 ± 1.3 Gy or 4.0 ± 1.4 Gy for IMPT_2F or IMPT_3F; the mean dose to the left ventricle resulted to 6.5 ± 1.6 Gy, 1.9 ± 1.5 Gy, 1.9 ± 1.6 Gy respectively. Similar sparing effects were observed for the liver, the kidneys, the stomach, the spleen and the bowels.

The EAR per 10,000 patients-years of secondary cancer induction resulted in 19.2 ± 5.7 for RA_RP and 6.1 ± 2.7 for IMPT_2F or 5.7 ± 2.4 for IMPT_3F. The RR for the left ventricle resulted in 1.5 ± 0.1 for RA_RP and 1.1 ± 0.1 for both IMPT sets. For the coronaries, the RR resulted in 1.6 ± 0.4 for RA_RP and 1.2 ± 0.3 for protons.

**Conclusion:**

With regard to cancer of the oesophagogastric junction type I and II, the use of intensity-modulated proton therapy seems to have a clear advantage over VMAT. In particular, the reduction of the heart and abdominal structures dose could result in an optimised side effect profile. Furthermore, reduced risk of secondary neoplasia in the lung can be expected in long-term survivors and would be a great gain for cured patients.

## Background

Oesophagal cancer is a particularly aggressive and common tumour entity with an estimated annual incidence of 572,000 new oesophagal cancer cases and 508,000 deaths worldwide in 2018 [1]. Despite curative intended multimodal treatment approaches, prognosis, especially in locally advanced tumours, remains poor. Oncological management of locally advanced oesophagal cancer depends on the histopathological findings and contains a diversity of multimodal treatment options. These include surgical resection, chemotherapy and radiotherapy. Neoadjuvant radiochemotherapy is regarded as the standard of care in squamous cell carcinoma. In adenocarcinoma, either perioperative chemotherapy or neoadjuvant radiochemotherapy can be performed [2–4]. The radiation dose, which is usually applied in the course of neoadjuvant radiochemotherapy, is mostly based on the protocol of the CROSS study with a total dose of 41.4 Gy in fractions of 1.8 Gy [5].

Due to the anatomic location of the oesophagus near to several organs at risk, such as the lungs, heart, spinal cord and stomach, the irradiation of oesophagal carcinomas is challenging. The complexity of the problem is in protecting as much as possible the nearby organs while maintaining adequate target coverage for the target volumes. There is a multitude of data from various studies that point out the pertinence of administered radiotherapy dose concerning cardiac and pulmonary complications [6, 7]. However, the introduction and establishment of novel irradiation techniques in radiotherapy led to an improvement of previously observed complications. In particular, a significantly lower incidence of pulmonary and cardiac late side effects could be achieved by the advancement from 3-dimensional conformal radiotherapy to intensity-modulated radiation therapy (IMRT) techniques [8, 9]. In this regard, Lin showed a significantly lower risk of cardiac- and noncancer-related death [10]. Xu [11] demonstrated on a cohort of 560 patients that heart and lung doses were independent predictors of overall survival as well as for organ-specific toxicity. These and other results allow the assumption that therapy-related side effects may have a significant influence on the overall prognosis of oesophagal cancer patients undergoing radiotherapy.

The use of volumetric modulated arc therapy (VMAT) was investigated by several authors [12–18] and, at least from a dosimetric point of view can be considered a promising further step in comparison to IMRT. Also, knowledge-based planning methods have been developed [19–22] to simplify the planning process.

The use of proton beam therapy (PBT) in the radio-oncological treatment of oesophagal carcinomas is the result of efforts to reduce the known toxicities further. Previously published dosimetric comparisons of PBT and IMRT indicate a considerable reduction of radiation exposure of organs at risk, especially regarding heart and lungs [23–25]. Welsh [26], in a dosimetric study, investigated the role of intensity-modulated proton therapy (IMPT) for advanced distal oesophagal tumors and showed a considerable reduction of the dose to the organs at risk compared to IMRT. Likewise, Shiraishi [27] demonstrated a significant decrease in radiation exposure to the whole heart and to the cardiac structures comparing PBT vs IMRT. Liu [25] compared at planning level the potential of VMAT versus IMPT for distal oesophagal cancer patients. The study concluded that protons resulted in dosimetrically preferable plans compared to VMAT. However, IMPT required careful tuning of the planning process to guarantee the needed robustness of the results. Yu [28] suggested a method based on the estimation of the water equivalent thickness to determine the more robust beam angles that are least affected by respiratory motion to increase plan robustness.

Concerning the sparing of lungs and cardiac structures with VMAT and IMPT, many studies addressed the issue also in different thoracic treatments. Ferris [29], for locally advanced non-small cell lung cancer and Scorsetti [30] and Baues [31] for Hodgkin’s lymphoma patients, demonstrated that both techniques could achieve significant sparing of these structures with a clear benefit when protons are considered.

Concerning the clinical outcome, Prayongrat [32] reported about 19 patients treated for oesophagal cancer with IMPT, presenting a complete response in 84% of the cases with an overall survival of 39.2 months (with a median follow up time of 17 months).

Lin [33] reported a phase IIB randomised trial on a cohort of 145 patients treated with IMRT (72 patients) or with PBT (73 patients). Of these, 107 were evaluated over a median follow-up time of 44.1 months and concluded that PBT reduced the risk and the severity of adverse events with an equivalent progression-free survival (51.2% at 3 years for PBT).

Sato [34] reported about concurrent chemotherapy with PBT for oesophagal squamous cell carcinoma patients. Over a cohort of 44 patients, the 3-year overall survival was 95.2%, and five patients needed endoscopic resection as salvage therapy due to local recurrence.

The primary aim of this in-silico planning study was to investigate the relative figure of merit of IMPT versus VMAT for advanced oesophagal cancer. The focus was set to the assessment of several appropriate dose-volume metrics for cardiac, abdominal and lung structures. Secondarily, the study aimed to estimate the risk of severe cardiac complications as coronary artery disease (CAD) or chronic heart failure (CHF) and the estimate of the excess absolute risk (EAR) of secondary cancer induction for the lungs.

## Materials and methods

### Patients selection, contouring and dose prescription

Twenty patients were selected for this retrospective in-silico planning study. All patients presented with advanced (cT3cNx cM0) adenocarcinoma of the gastro-oesophageal junction (AEG) and were treated with neoadjuvant radiochemotherapy analogue to the CROSS-regime and concomitant weekly chemotherapy with carboplatin and Paclitaxel (50 mg per square meter of body-surface area) [5]. AEG were classified according to Siewert with AEG type I as carcinomas of the distal oesophagus and AEG type II as carcinomas of the true cardia [35].

The gross tumour volume (GTV) was identified on the pre-chemotherapy extent of the disease using the initial PET/CT scan, endoscopy report and CT scan. The entire oesophagal wall, including any disease that extended through the wall, was contoured as the GTV as well as any PET/CT-avid or enlarged lymph nodes. The clinical target volume (CTV) encompassed the peri-oesophagal lymph nodes, mediastinal lymph nodes and the submucosal spread longitudinally along the oesophagus. This required a 3–4 cm expansion on the GTV superiorly and inferiorly and a 1.0–1.5 cm radial expansion. The planning target volume (PTV) was generated adding 0.7 cm isotropically. The same CTV was used for both proton and photon planning. The PTV was used for the photon optimisation. To note that for protons, the PTV was used only for dose reporting purposes since the robust optimisation was based on the CTV as discussed below. The following organs at risk (OAR) were segmented and considered: the lungs, the whole heart with its structures (atrial and ventricular left and right chambers and the coronaries), the oesophagus, the liver, the kidneys, the spleen, the stomach, the bowels and the spinal canal. For the present study, cardiac substructures were retrospectively contoured for the selected 20 patients using a heart atlas [36]. All segmentations were executed on planning CT with 3 mm slice thickness.

The dose prescription was 41.4 Gy in 23 daily fractions as in the clinical routine. All the plans were normalised to 100% as the mean dose to the PTV; the dose-volume objectives for the target volumes and organs at risk are summarised in Tables 1 and 2.

**Table 1:** Summary of the planning objectives and average results (uncertainty expressed as 1 standard deviation) for the gross target volume (GTV), the clinical target volume (CTV) and for the planning target volume (PTV) together with the presence of statistical significance difference among any couple of datasets

	Objective	RA_RP	IMPT_3F	IMPT_2F	p
**GTV**					
**Mean [Gy]**	41.4	41.5 ± 0.4	41.4 ± 0.4	41.4 ± 0.4	–
**D**_**1%**_**[Gy]**	Minimize	42.2 ± 0.2	42.4 ± 0.3	42.4 ± 0.3	–
**V**_**98%**_**[%]**	≥98	99.7 ± 1.1	99.0 ± 1.3	98.2 ± 2.1	B,C
**CTV**					
**Mean [Gy]**	41.4	41.6 ± 0.1	41.4 ± 0.1	41.4 ± 0.1	–
**D**_**1%**_**[Gy]**	Minimize	42.4 ± 0.2	42.5 ± 0.3	42.5 ± 0.3	–
**V**_**98%**_**[%]**	≥98	99.5 ± 0.6	98.5 ± 1.1	97.8 ± 1.8	B,C
**PTV**					
**Mean [Gy]**	41.4	41.4 ± 0.0	41.4 ± 0.0	41.4 0.0	–
**D**_**1%**_**[Gy]**	Minimize	42.7 ± 0.3	42.8 ± 0.2	42.8 ± 0.2	–
**V**_**98%**_**[%]**	≥90	89.9 ± 1.2	93.9 ± 1.7	94.1 ± 2.0	B,C
**V**_**95%**_**[%]**	≥95	97.2 ± 0.7	98.3 ± 0.7	98.4 ± 0.7	B,C
**HI [%]**	minimize	5.6 ± 0.6	4.5 ± 0.8	4.4 ± 0.9	B,C
**CI**_**Paddick**_	minimize	0.88 ± 0.01	0.89 ± 0.02	0.89 ± 0.02	–

P: Statistical significance: A: IMPT_2F vs IMPT_3F; B: IMPT_2F vs RA_RP; C: IMPT_3F vs RP_RP

*D*_*x*_ Dose received by x volume, *V*_*x*_ Volume receiving x dose, *CI*_*Paddick*_ Conformity index, *HI* Homogeneity index, *RA_RP* RapidArc Volumetric modulated arc therapy with RapidPlan optimization, *IMPT* Intensity modulated proton therapy with robust optimization (2 fields: _2F; 3 fields: _3F)

**Table 2:** Summary of the planning objectives and average results (uncertainty expressed as 1 standard deviation) for the main organs at risk investigated in the study) together with the presence of statistical significance difference among any couple of datasets

	Objective	RA_RP	IMPT_2F	IMPT_3F	p
**Lungs**					
**Mean [Gy]**	≤12Gy	8.6 ± 2.9	3.2 ± 1.5	2.9 ± 1.2	A,B,C
**V**_**20Gy**_**[%]**	≤20%	10.5 ± 5.6	6.9 ± 4.2	5.9 ± 2.6	A,B,C
**Whole heart**					
**Mean [Gy]**	≤10Gy	9.9 ± 1.9	3.7 ± 1.3	4.0 ± 1.4	B,C
**V**_**30Gy**_	≤10%	5.9 ± 2.9	4.9 ± 2.2	4.9 ± 2.3	B,C
**Left ventricle**					
**Mean [Gy]**	Mimimise	6.5 ± 1.6	1.9 ± 1.5	1.9 ± 1.6	B,C
**D**_**0.1cm3%**_**[Gy]**		23.9 ± 9.2	25.2 ± 10.9	25.1 ± 11.4	B,C
**Left Anterior Descending artery**					
**Mean [Gy]**	Mimimise	3.1 ± 0.5	0.1 ± 0.1	0.1 ± 0.2	B,C
**D**_**0.1cm3**_**[Gy]**	Mimimise	5.3 ± 1.1	0.9 ± 2.4	3.8 ± 5.9	A,B,C
**Left coronary artery**					
**Mean [Gy]**	Mimimise	6.2 ± 4.2	1.5 ± 3.4	2.9 ± 4.7	A,B,C
**D**_**0.1cm3**_**[Gy]**	Mimimise	10.9 ± 14.1	6.5 ± 15.5	8.0 ± 15.4	A,B,C
**Liver**					
**Mean**	≤15Gy	11.5 ± 1.8	3.8 ± 1.8	3.6 ± 1.2	B,C
**Left kidney**					
**Mean [Gy]**	≤15Gy	3.4 ± 1.9	2.1 ± 1.9	1.7 ± 1.5	A,B,C
**V**_**20Gy**_**[%]**	≤32%	1.8 ± 2.2	1.7 ± 1.7	0.9 ± 1.0	A
**Right kidney**					
**Mean [Gy]**	≤15Gy	2.9 ± 2.1	1.6 ± 1.8	1.2 ± 1.3	A,B,C
**V**_**20Gy**_**[%]**	≤32%	1.1 ± 2.2	1.9 ± 3.1	0.4 ± 0.9	A,C
**Stomach**					
**Mean [Gy]**	Mimimise	8.5 ± 6.9	3.4 ± 6.9	3.5 ± 6.7	B,C
**D**_**1%**_**[Gy]**	Mimimise	31.3 ± 11.1	28.8 ± 14.0	28.8 ± 13.6	B,C
**D**_**1cm3**_**[Gy]**	Mimimise	32.5 ± 11.5	30.7 ± 14.1	30.7 ± 13.6	B,C
**Spleen**					
**Mean [Gy]**	Mimimise	8.2 ± 2.2	4.6 ± 4.0	2.7 ± 3.1	A,B,C
**D**_**1%**_**[Gy]**	Mimimise	23.4 ± 7.6	22.9 ± 11.3	21.5 ± 8.5	C
**Bowel**					
**Mean [Gy]**	Mimimise	5.6 ± 3.5	0.8 ± 0.9	1.4 ± 1.49	A,B,C
**D**_**1%**_**[Gy]**	Mimimise	17.7 ± 9.6	14.9 ± 11.3	16.2 ± 10.7	–
**D**_**1cm3**_**[Gy]**		19.8 ± 11.2	19.4 ± 12.7	20.1 ± 12.1	A
**Spinal canal**					
**D**_**1%**_**[Gy]**	Mimimise	16.6 ± 2.1	22.2 ± 4.9	12.3 ± 1.7	A,B,C
**D**_**0.1cm3%**_**[Gy]**	Mimimise	17.0 ± 2.2	23.5 ± 4.6	13.2 ± 1.8	A,B,C

P: Statistical significance: A: IMPT_2F vs IMPT_3F; B: IMPT_2F vs RA_RP; C: IMPT_3F vs RP_RP

*D*_*x*_ Dose received by x volume, *V*_*x*_ Volume receiving x dose, *RA_RP* RapidArc Volumetric modulated arc therapy with RapidPlan optimization, *IMPT* Intensity modulated proton therapy with robust optimization (2 fields: _2F; 3 fields: _3F)

### Photon planning

The photon plans were optimised according to the volumetric modulated arc therapy technique (RapidArc, RA) implemented for a TrueBeam linear accelerator (Varian Medical Systems, Palo Alto, USA). Flattening filter free photon beams (beam quality of 6MV) were used for the study. The Photon Optimiser algorithm of the Eclipse treatment planning (version 16.0) system was used for the inverse planning phase, and the final dose calculation was done with the Acuros-XB algorithm. The calculation grid was set to 2.5 mm. For all plans, the beam arrangement was defined according to a class solution consisting of two full arcs with collimator angle set to 10–350°. The first set of RA plans was optimised and used to train a knowledge-based predictive model with the RapidPlan (RP) engine of Eclipse (similarly to [22]). All cases were further re-optimised using the RP model (RA_RP) to further push the consistency of the results [37].

### Proton planning

The intensity-modulated proton therapy (IMPT) plans were designed and optimised for the ProBeam proton system (Varian Medical Systems, Palo Alto, USA) using the beam spot scanning technique. The beam data are derived from the commissioning of the Scripps Proton Center (San Diego, California, USA), constitute the reference beam data in Eclipse and were used with permission. The dose distribution optimisation was performed using the Nonlinear Universal Proton Optimiser (v16.0). The final dose calculation, the Proton Convolution Superposition algorithm (v16.0) was used with a grid size of 2.5 mm and a constant relative biological effectiveness RBE of 1.1.

All patients were planned with two standardised class solution: i) two fields arrangement (IMPT_2F) with two posterior oblique fields (140–215°) and ii) three fields arrangement (IMPT_3F) with an additional anterior field (0°). Small, individualised gantry angles tuning was allowed, according to the target position, to minimise the healthy tissue involvement.

The CTV was subject to robust optimisation methods to account for setup and range uncertainties considering ±4 mm shifts in the isocentre along the x-y-z coordinates and ± 3% in beam range. The 4 mm shifts are not to be intended as a proton specific margin to the CTV but the positioning uncertainty of it. The robust optimisation should result in plans minimising the trade-offs derived from the applied uncertainties, as discussed in [38].

### Quantitative assessment of dose-volume metrics

The numerical analysis of the dose distributions was performed employing several V_x_ and D_x_ parameters (V_x_ represents the volume receiving at least an x level of dose (in % or in Gy) and D_x_ is the minimum dose that covers an x fraction of volume (in % or in cm^3^) [39] derived from the dose-volume histograms (DVH) and used as quantitative metrics.

For the PTV, the homogeneity index (HI) measured the variance of the dose and was defined as HI = (D_5%_-D_95_)/D_mean_. The Paddick Conformity Index, CI_Paddick_ [40], was defined by selecting the 98 isodose as the reference. The average DVHs were computed, for each structure, with a dose binning resolution of 0.02Gy. Proton doses are reported in Cobalt equivalent Gy (corrected for the 1.1 RBE factor).

The statistical significance of the differences between the various datasets was computed employing the Wilcoxon matched-paired signed-rank test with a threshold to significance set to 0.05 with the SPSS package (version 22, IBM Corporation, Armonk, USA).

### Modelling the risk of toxicity and secondary cancer induction

The estimation of the relative risk (RR) of cardiac complications was performed for the coronaries and the left ventricle chamber. The endpoint was disease or failure, and the calculations were performed according to the linear model proposed by van Nimwegen [41, 42] for Hodgkin lymphoma patients. This model correlated the coronary heart disease to the mean dose to the heart and was based on the observations of Darby [43] for breast cancer patients. The excess relative risk was fixed to 7.4 and 9.0% per Gy, respectively for the coronaries and for the left ventricle. To mention that all the data were based on photon treatments and therefore possibly not strictly applicable to the proton case. The choice of this model is consistent with other similar investigations [44] and reasonably valid in the absence of definitive data.

The excess absolute risk of secondary malignancy induction (EAR) for any specific organ (*org)* was defined as described in [45]:

$$ {EAR}^{org}=\mu\ \frac{1}{V_T}\sum \limits_iV\left({D}_i\right) RED\left({D}_i\right) $$

where *V*_*T*_ is the total organ volume. The sum is over all the bins of the differential DVH, *V(D*_*i*_*)* is the absolute volume receiving a dose *D*_*i*_. μ is the slope of cancer induction based on the atomic bomb survivors’ data [46] corrected for the age distribution. The value used in this analysis was 3.78. *RED(D)* is the dose-response, modelled to fit the Hodgkin’s patient’s data group [46]. The organ equivalent dose (OED): $OED=\frac{1}{V_T}{\sum}_iV\left({D}_i\right) RED\left({D}_i\right)$ was introduced as the dose in Gray, which, when uniformly distributed, causes the same radiation-induced cancer incidence. The so-called full model [47] was applied in the present study and includes all the biological aspects of cell killing, repopulation/repair, and fractionation:

$$ OED=\frac{1}{V_T}\sum \limits_iV\left({D}_i\right)\ \frac{e^{-{\alpha}^{\prime }{D}_i}}{\alpha^{\prime }R}\ \left(1-2R+{R}^2{e}^{\alpha^{\prime }{D}_i}-{\left(1-R\right)}^2\ {e}^{-\frac{\alpha^{\prime }R}{1-R}\ {D}_i}\right) $$

where *R* is the parameter accounting for repopulation and/or repair and models the ability of the tissue to recover between two fractions (ranging from 0 for no recovery to 1 for full recovery). In the present study, R was set to 0.83, (the lungs were jointly considered as a single structure); α’ was set to 0.042Gy^− 1^ [46].

## Results

### Dosimetric comparison

**Fig. 1:**
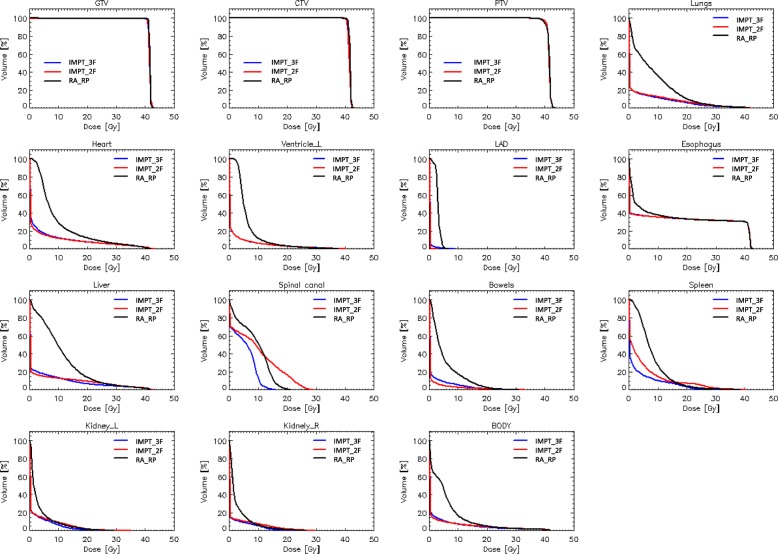
Average dose-volume histograms for the target volumes and the main organs at risk investigated. In the figure the left or right structures are labelled with the _L or _R suffix; the left anterior descending coronary is labelled LAD

Figure 1 presents the average dose-volume histograms for the three techniques, for all the target volumes and the main organs at risk considered in the study. From a qualitative point of view, the target volumes resulted in equivalent coverage. The IMPT plans presented an average better sparing effect over the dose range 0 to 20Gy. The average DVH for the spinal canal and the spleen is worse for the IMPT_2F group of plans due to the geometrical arrangement of the fields with respect to these organs, the absence of the anterior field requires more contribution from the posterior ones.

The quantitative analysis of the DVHs is summarised in Table 1 for the GTV, CTV and PTV, and in Table 2 for the OARs.

Regarding target volumes, all three techniques met the planning objectives on average. V_98%_ exceeded 98% for both GTV and CTV, while V_95%_ was greater than 95% for the PTV. Dose homogeneity resulted better than 5% for both proton groups while it was slightly worse (5.6 ± 0.6%) for photons. The conformality of the dose distributions resulted equivalent for all approaches.

For all the OARs statistically significant differences were observed for most of the metrics between photons and protons and for some parameters also between IMPT_2F and IMPT_3F mostly because the absence of the anterior field unbalanced the dose distributions towards the posterior region. This was particularly relevant for the spleen and the spinal canal. In general, photon and proton plans met the planning aims for all the structures with quantitative constraints. Nevertheless, the mean dose to the lungs, to the whole heart and the left ventricle as well as the mean dose to the arteries resulted in a substantial improvement for the protons with relative gains ranging from a factor of ca. 2.2 to 3.3 (up to about 30 for the left anterior descending artery). A similar significant advantage was observed for the abdominal structures.

Smaller differences, although mostly statistically significant, were observed for the near-to-maximum doses or for metric scored above 20Gy. In the high-dose domain, the observed differences between protons and photons were modest.

### Toxicity and secondary cancer risk estimates

Table 3 reports the RR estimates for the whole heart, for the left ventricle chamber and the entire coronary volume. The uncertainties reported are relative to the inter-patient variance.

**Table 3:** Estimates of the relative risk of cardiac failure and Excess absolute risk (EAR) (per 10,000 patient-years) of secondary cancer induction estimated with the full model. Results are shown as averages (with uncertainty expressed as 1 standard deviation). The *p* value is relative to the Wilcoxon signed rank paired test

Organ	Model	Endpoint	RA_RP	IMPT_2F	IMPT_3F	P
**Whole heart**	RR	CHF	1.7 ± 0.1	1.3 ± 0.1	1.3 ± 0.1	B,C
**Left Ventricle**	RR	CHF	1.5 ± 0.1	1.1 ± 0.1	1.1 ± 0.1	B,C
**Coronaries**	RR	CAD	1.6 ± 0.4	1.2 ± 0.3	1.2 ± 0.3	A,B,C
**Lungs**	EAR	RSCI	19.2 ± 5.7	6.1 ± 2.7	5.7 ± 2.4	A,B,C

*RR* Relative risk, *CAD* Coronay artery disease, *CHF* Chronic heart failure, *EAR* Excess absolute risk, *RSCI* Risk of secondary cancer inducton, *RA_RP* RapidArc Volumetric modulated arc therapy with RapidPlan optimization, *IMPT* Intensity modulated proton therapy with robust optimization (2 fields: _2F; 3 fields: _3F). Statistical significance: A: IMPT_2F vs IMPT_3F; B: IMPT_2F vs RA_RP; C: IMPT_3F vs RP_RP

Based on these results, the findings from proton plans are suggestive of a remarkable and significant sparing effect between photons and protons for both the endpoints (CAD and CHF) with a highly significant average reduction of the RR of 0.4 for both CHF and CA.

The analysis of the EAR (expressed in cases per 10,000 patient-years) was restricted to the combined lungs. It showed a macroscopic and systematic highly significant reduction of the risk for IMPT with respect to RA. In particular, the average EAR gain (defined as the EAR for photons – the EAR for protons) resulted in 13.1 and 13.5 cases per 10,000 patients-year for IMPT_2F and IMPT_3F compared to RA_RP.

## Discussion

The present study aimed at comparing in-silico VMAT and IMPT plans for a cohort of 20 advanced-stage tumours of the oesophago-gastric junction and to determine from dosimetric metrics and risk assessments the relative merits of the two treatment modalities. Although the evaluation of VMAT and IMPT for oesophagal cancer was already explored at planning level by some authors, with the present investigation, we aimed at incorporating predictive models for risk assessment to the standard dosimetric methods. In our analysis, we proved that the use of IMPT, compared to VMAT, might lead to a significant reduction in the risk of OAR damage. In addition, we evaluated our results with regard to the relative risk of cardiac side effects. Also, we saw a significant advantage in the use of IMPT compared to RA-RT. Two different beam arrangements were considered for IMPT as a basic attempt to appraise the impact of the beam geometry on the chosen metrics. Robust optimisation was applied to the IMPT plans to account for uncertainties in the range calibration and in the position of the patients. Minimal clinical evidence exists about the role of IMPT versus intensity-modulated photons and the data published are either from mono-institutional non randomised studies on small cohorts [32, 34, 48] or from randomised studies allowing for mixed techniques. As an example, the Lin phase II trial [33] allowed either IMRT or VMAT for the photon treatments or passive scattering or IMPT for the protons. In the absence of definitive clinical evidence, planning studies are still needed to better define the field of investigation.

From a dosimetric perspective, the study of Xu [11] found that a mean lung dose of 10Gy and for the heart a V_30Gy_ of 45% were the thresholds best separating the sub-cohorts with or without radiation-induced cardiac and pulmonary complications with a direct impact on worse survival. In our study, both thresholds were respected for VMAT and IMPT with a statistically significant improvement for the IMPT plans. Protons allowed a reduction of the mean dose to the lungs of a factor of about three compared to VMAT while V_30Gy_ for the heart never reached 6.0 Gy.

The observed dosimetric gain with IMPT should be translated into a reduced risk of severe complications or secondary cancer induction. Concerning the heart, the Nimwegen-Darby models were applied [41–43]. It is important to mention, preliminarily that those studies were based on photon treatments and, therefore, the models might not be strictly applicable also to protons. In their research, Levis [43] proposed to consider the entire volume of the coronaries as the structure of reference for the assessment of the relative risk of CAD. A similar approach was already performed in the study of Scorsetti [30], and this was the model also followed in the present study. Although the choice is somehow arbitrary, in the absence of proven and consolidated models, it is considered to be a reasonable working hypothesis. For the heart, we studied both the whole organ as well as the left ventricle and assessed the relative risk of chronic failure for both. The results achieved by Levis and Scorsetti (for lymphoma patients) presented a RR for CAD ranging from 1.4 ([30] for the LAD only) to 2.2 [43] for VMAT plans. The results of the present investigation resulted in a RR for CAD for the coronaries of 1.6 ± 0.4. Protons would allow a further relative gain of 0.4 (− 25% with respect to the RR for RA_RP) with a strong statistical significance of the finding. Considering the relative risk of CHF, Levis and Scorsetti [30, 44] reported values ranging from 1.3 to 1.4 for the left ventricle or the whole organ while in the case of oesophagal cancer, the anatomical proximity of the target to the cardiac structures implied a slightly higher estimation of the RR to 1.5 (left ventricle) or 1.7 (whole heart) for VMAT plans. The use of protons would have reduced the RR by 0.4, i.e.-23, − 33% respectively with respect to the RA_RP. All these studies suggest a largely reduced risk of cardiac disease from the use of IMPT when compared to state-of-the-art photon-based VMAT therapy.

The assessment of the risk of secondary cancer was limited to the estimates for the lungs. Scorsetti [30] reported an EAR of 22.6 and 15.3 for VMAT and IMPT, respectively. In the present study, the EAR for VMAT was consistent (19.2±) while for IMPT the EAR in the case of the oesophagal patients resulted in 5.7–6.1 depending on the number of fields used. This means a relative reduction of the EAR by a factor of about 3 when moving from VMAT to IMPT.

The results of the present study suggest that the beam geometry chosen for IMPT is not severely affecting the dose-volume metrics nor the risk estimates, also for the organs like spinal cord or spleen where differences were observed. It is, of course, clear that “reasonable” angles should be selected but that simple class solution could be implemented for the global population. Yu [28] suggested considering water equivalent thickness analysis to identify the beam angles least affected by respiratory motion as a first approach to plan robustness. The present study implemented positioning uncertainties (4 mm) during the optimisation as a more advanced and automated tool to account for the global problem. In addition to robust optimisation, it would be advisable to consider, if technically doable, to implement respiratory gating methods, e.g. breath-hold, to further mitigate the respiration induced uncertainties.

The combination of the risk estimates for cardiac failure and secondary cancer induction is strongly suggestive about the relevance of IMPT in the radiation treatment of oesophagal patients.

Among the limitations of the study, the rather small sample size shall be disclosed. Although standard for planning investigations, the relatively small cohort investigated does not guarantee a comprehensive scoring of the spectrum of possible cases. The extension of the disease, the inter-patient anatomical variation implies a certain variance or uncertainty in the average results reported. This can be estimated to be ranging from about 10% or less to 50% (e.g. in the stomach) or more with respect to the reported mean values (Table 2) for the structures with large variability among patients.

A second potential concern is linked to the use of cardiac substructures for the risk modelling and the use of these segmented structures during the plan optimisation. In the present study, an atlas-based approach was followed. The Feng atlas [15] was used as in the Levis [43] study. It is obvious that the image quality of standard planning CT is sub-optimal for accurate heart segmentation and that motion-induced artefacts have an impact on the process. Nevertheless, it is crucial to acquire the knowledge and to raise awareness about the relevance and the value of the segmentation of the individual cardiac elements in addition to the whole organ. Dedicated cardiac scanning and/or time-resolved acquisitions synchronised with the heartbeat rate might deserve future systematic investigation.

A potential bias in the study might derive from the use of a constant 1.1 RBE for the protons. A variable RBE with some associated uncertainty would be more realistic with some impact on tumour control and normal tissue complication probabilities. In a recent review, McNamara [49] concluded that the use of fixed RBE is simplistic and might raise concerns for treatment planning decisions. Inclusion of variable RBE tools would be a fundamental advance in treatment planning systems but at the current stage is acknowledged as a limit of the present investigation.

Concerning the quality of the treatment plans compared in the study, the use of the knowledge-based RapidPlan engine for photons might have introduced a bias in favour of the photon plans compared to the manually optimised IMPT plans. We aimed to minimise this risk paying meticulous attention to the IMPT plans. In future, the availability of RapidPlan also for protons [50] might further reduce this potential bias, and we aim to investigate this aspect as a future step in the research program.

## Conclusion

With regard to cancer of the oesophago-gastric junction type I and II, the use of intensity-modulated proton therapy showed to have a clear advantage over volumetric modulated arc therapy with photon therapy. In particular, the reduction of the heart and abdominal structures dose could result in an optimised side effect profile. Furthermore, a reduced risk of secondary neoplasia in the lung can be expected in long-term survivors and would be a great gain for cured patients.

## Data Availability

The data are available upon request.

## Abbreviations

AEG: Adenocarcinoma of the oesophago-gastric junction
CAD: Coronary artery disease
CHF: Chronic heart failure
CI: Conformity index
CT: Computed tomography
CTV: Clinical target volume
DVH: Dose-volume histogram
EAR: Excess absolute risk
GTV: Gross tumour volume
HI: Homogeneity index
IMPT: Intensity-modulated proton therapy
IMRT: Intensity-modulated radiation therapy
LAD: Left anterior descending coronary
LV: Left ventricle
MV: Megavoltage
OAR: Organ at risk
OED: Organ equivalent dose
PET: Positron emission tomography
PBT: Proton beam therapy
PTV: Planning target volume
RA: RapidArc
RP: RapidPlan
RBE: Relative biological effectiveness
RR: Relative risk
VMAT: Volumetric modulated arc therapy
